# Composite NiCo_2_O_4_@CeO_2_ Microsphere as Cathode Catalyst for High‐Performance Lithium–Oxygen Battery

**DOI:** 10.1002/advs.202200523

**Published:** 2022-04-27

**Authors:** Yuanhui Wu, Haoran Ding, Tianlun Yang, Yongji Xia, Hongfei Zheng, Qiulong Wei, Jiajia Han,, Dong‐Liang Peng, Guanghui Yue

**Affiliations:** ^1^ State Key Lab of Physical Chemistry of Solid Surface Fujian Key Laboratory of Materials Genome Collaborative Innovation Center of Chemistry for Energy Materials College of Materials Xiamen University Xiamen 361005 P. R. China

**Keywords:** DFT calculations, lithium–oxygen batteries, porous, rare earth metal oxide, transition metal oxide

## Abstract

The large overpotential and poor cycle stability caused by inactive redox reactions are tough challenges for lithium–oxygen batteries (LOBs). Here, a composite microsphere material comprising NiCo_2_O_4_@CeO_2_ is synthesized via a hydrothermal approach followed by an annealing processing, which is acted as a high performance electrocatalyst for LOBs. The unique microstructured catalyst can provide enough catalytic surface to facilitate the barrier‐free transport of oxygen as well as lithium ions. In addition, the special microsphere and porous nanoneedles structure can effectively accelerate electrolyte penetration and the reversible formation and decomposition process of Li_2_O_2_, while the introduction of CeO_2_ can increase oxygen vacancies and optimize the electronic structure of NiCo_2_O_4_, thereby enhancing the electron transport of the whole electrode. This kind of catalytic cathode material can effectively reduce the overpotential to only 1.07 V with remarkable cycling stability of 400 loops under 500 mA g^−1^. Based on the density functional theory calculations, the origin of the enhanced electrochemical performance of NiCo_2_O_4_@CeO_2_ is clarified from the perspective of electronic structure and reaction kinetics. This work demonstrates the high efficiency of NiCo_2_O_4_@CeO_2_ as an electrocatalyst and confirms the contribution of the current design concept to the development of LOBs cathode materials.

## Graphical abstract

The NiCo_2_O_4_@CeO_2_ electrocatalyst possesses a special porous urchin‐like structure. It exhibits excellent electrochemical performance in Li–O_2_ batteries. The overpotential is only 1.07 V and it has a highly long life of 400 cycles under a current density of 500 mA g^−1^. In addition, the enhanced mechanism of electrochemical performance is clarified by density functional theory calculations.

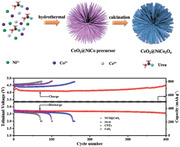

## 1 Introduction

The growing requirement for future energy storage systems has promoted the rapid development of battery research.^[^ ^1^, ^2^, ^3^, ^4^ ^]^ Among them, nonaqueous rechargeable lithium–oxygen batteries (LOBs) with ultrahigh theoretical energy density (around 3505 Wh kg^−1^) stand out among numerous rechargeable batteries, showing unprecedented potential.^[^ ^5^, ^6^, ^7^, ^8^ ^]^ LOBs have widespread applications in transportation, portable electronic devices, and energy storage applications, which have attracted much attention.^[^ ^9^, ^10^, ^11^, ^12^, ^13^ ^]^ The typical discharge reaction (oxygen reduction reaction (ORR)) of LOBs is: 2Li + O_2_ ↔ Li_2_O_2_, while the charge reaction (oxygen evolution reaction (OER)) is the decomposition of Li_2_O_2_.^[^ ^14^, ^15^, ^16^ ^]^ However, the insoluble and insulating products generated during the discharge process are easy to deposit around the positive electrode material and block the active site, resulting in excessive overpotential,^[^ ^17^, ^18^ ^]^ poor stability, short cycle life,^[^ ^19^ ^]^ and even final death of batteries.^[^ ^20^ ^]^ Therefore, the positive electrode material should not only have a high specific surface area to accommodate Li_2_O_2_, but also a high catalytic activity to promote the decomposition of lithium peroxide.^[^ ^21^ ^]^ The development of bi‐functional electrocatalysts that can effectively enhance OER and ORR is one of the challenges of high‐powered LOBs.^[^ ^22^ ^]^

Currently, bi‐functional catalysts for LOBs mainly include carbon‐based materials, noble metals, transition metal oxides, etc.^[^ ^23^, ^24^ ^]^ Although noble metals such as Pt/C have been proved to be effective catalysts, the disadvantage of high prices makes people more inclined to use transition metal oxides with low cost, abundant reserves, and relatively high electrochemical activity.^[^ ^25^, ^26^ ^]^ In particular, the spinel NiCo_2_O_4_ has received widespread attention due to its extremely low overpotential and corrosion resistance.^[^ ^27^ ^]^ Some studies have shown that NiCo_2_O_4_, as a bimetallic oxide, can utilize the synergistic effect between Ni and Co to promote OER and ORR^[^ ^28^, ^29^ ^]^ and it is also more conductive than single metal oxide. Besides, NiCo_2_O_4_ with different morphology would help to reduce discharge/charge overpotential and increase capacity. Several different types of nanostructured NiCo_2_O_4_, including nanoparticles,^[^ ^30^ ^]^ nanoplates,^[^ ^31^ ^]^ nanosheets,^[^ ^32^ ^]^ nanowires,^[^ ^33^ ^]^ and microspheres,^[^ ^34^, ^35^ ^]^ have been successfully synthesized and applied in various energy storage devices. However, compared with other reported high‐efficiency cathodes, the cathode structure and catalytic effect of NiCo_2_O_4_ still need to be further optimized and strengthened.^[^ ^36^ ^]^

Cerium dioxide (CeO_2_) possesses high catalytic activity as a rare earth metal oxide. It can be used as an “oxygen buffer” to flexibly change the valence state according to the concentration of oxygen, thus helping to enhance the migration ability of oxygen species and significantly improve the ORR performance of LOBs.^[^ ^37^, ^38^, ^39^ ^]^ This process can be expressed as CeO_2_↔CeO*_x_* + (2−*x*)/2O_2_ (1.5 < *x* < 2).^[^ ^40^, ^41^ ^]^ The oxygen in the lattice of CeO_2_ has high activity and fluidity, which is conducive to the generation of oxygen vacancies, thereby increasing the active sites of the reaction. Studies have shown that the introduction of CeO_2_ will increase the specific capacity of LOBs, reduce overpotential, and improve cyclability.^[^ ^42^, ^43^, ^44^, ^45^, ^46^ ^]^ However, the poor conductivity and minimal area of pure CeO_2_ nanoparticles limit their development, which needs to be combined with a conductive substrate with a high specific surface area.^[^ ^47^, ^48^, ^49^ ^]^ Therefore, compositing the urchin‐like NiCo_2_O_4_ with CeO_2_ nanoparticles is a feasible solution to significantly promote the performance of LOBs.

In this paper, we report a fairly convenient approach to directly synthesize a superior composite structure with NiCo_2_O_4_ urchin‐like microsphere as the core and CeO_2_ nanoparticles as an embellishment, working as an effective OER and ORR dual‐function catalyst for the rechargeable nonaqueous LOBs. Such microscale structure can provide sufficient catalytic surface and open space to facilitate the transfer of oxygen or lithium ions, provide more active sites and adequate specific surface area to store the products during the discharge process. In addition, the porous structure of the nanoneedles significantly promotes barrier‐free oxygen transmission and electrolyte penetration, helping to establish multi‐dimensional charge diffusion paths. The introduction of CeO_2_ can increase oxygen vacancies, which not only generate a great number of nonsaturable sites, but also erect an electron transport bridge, thereby highly promoting the charge transfer kinetics. The excellent catalytic effect of the composite material on the OER and ORR of the LOBs is mainly attributed to these synergistic advantages, which can effectively reduce overpotential to 1.07 V and extend the cycle life that can reach 400 times under the restricted capacity of 500 mA h g^−1^ with an ampere density of 500 mA g^−1^. This special urchin‐like composite structure provides a new way to build up the performance of LOBs.

## 2 Results and Discussion

### 2.1 Morphology and Structure Characterization

**Figure 1:**
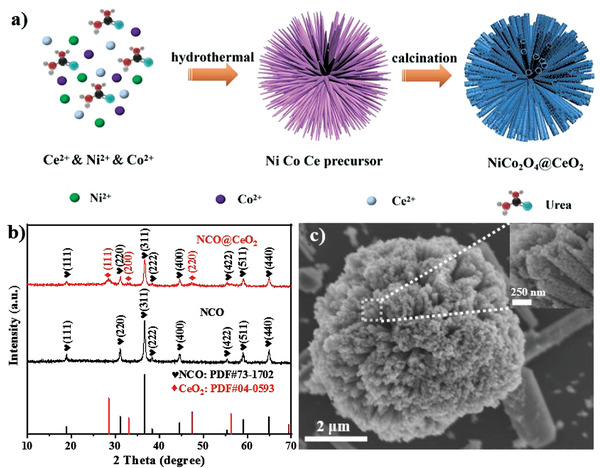
a) The schematic diagram of preparation process for NiCo_2_O_4_@CeO_2_. b) XRD patterns of NiCo_2_O_4_@CeO_2_ and NiCo_2_O_4_. c) SEM images of NiCo_2_O_4_@CeO_2_ at low magnification (inset: enlarged image of nanoneedles).

The synthesis method of composite urchin‐like NiCo_2_O_4_@CeO_2_ microspheres is achieved by a two‐step method, as shown in **Figure** **1**a. First, the NiCo_2_O_4_@CeO_2_ precursor is prepared by a one‐step hydrothermal reaction. After being calcined in air, the precursor changes from smooth nanoneedles to porous nanotubes due to unbalanced diffusion,^[^ ^50^ ^]^ which is denoted as NiCo_2_O_4_@CeO_2_ in this work. For comparison, NiCo_2_O_4_ microspheres and CeO_2_ nanoparticles are also prepared by the same procedure, respectively.

The phase structure and composition of the synthesized samples were determined by X‐ray diffraction (XRD) characterization. The XRD pattern of the precursor (Figure S1, Supporting Information) shows that it is composed of Ni_2_(OH)_2_CO_3_·4H_2_O, Co(CO_3_)_0.5_(OH)·0.11H_2_O, and Ce(CO_3_)_2_O·H_2_O. From Figure 1b, it could be found that in the crystal structure of all composite materials, all typical peaks can be exactly matched with spinel NiCo_2_O_4_ (JCPDS no. 73–1702) and CeO_2_ (JCPDS no. 04–0593). Specifically, the typical diffraction peaks located at 18.9°, 31.1°, 36.7°, 38.3°, 44.6°, 55.4°, 59.1°, and 64.9° can be accurately indexed as (111), (220), (311), (222), (400), (422), (511), and (440) planes of NiCo_2_O_4_, and the three characteristic peaks at 28.5°, 33.0°, and 47.4° can be ascribed to (111), (200), and (220) planes of CeO_2_. The structure of individual CeO_2_ and its precursor are demonstrated in Figure S2 (Supporting Information).^[^ ^51^, ^52^, ^53^ ^]^ It can be confirmed that the hydroxy carbonate of the precursor is well converted into oxide, and the NiCo_2_O_4_@ CeO_2_ composite structure is successfully formed. It should be noted that compared with a single substance, the diffraction peak intensity of NiCo_2_O_4_ decreases with the recombination of CeO_2_, indicating a slight decrease in crystallinity. In any case, these results confirm that NiCo_2_O_4_ and CeO_2_ coexist in the composite structure, and the prepared samples have high crystallinity without impurities.

The morphology of NiCo_2_O_4_@CeO_2_, NiCo_2_O_4_, and carbon nanotubes (CNTs) was detected by scanning electron microscope (SEM) in Figure 1c and Figure S3 (Supporting Information). Figure S4a (Supporting Information) shows that the precursor of the composited NiCo_2_O_4_@CeO_2_ is about 4 µm urchin‐like microsphere, each of which has sharp and smooth nanoneedles with a length of 2 µm (Figure S4b, Supporting Information). After being calcined in the air atmosphere, the entire urchin‐like sphere swells to about 5 µm (Figure 1c). Meanwhile, the precursor changes from smooth nanoneedles to porous and rough nanotubes due to nonequilibrium diffusion,^[^ ^50^ ^]^ and each nanotube is about 250 nm thick, as shown in the inset of Figure 1c. There is no significant difference in the morphology of NiCo_2_O_4_ and NiCo_2_O_4_@CeO_2_ because they are both urchin‐like microspheres, as seen in Figure S3a,b (Supporting Information). The only difference between them may be the adsorbed CeO_2_ nanoparticles on the surface. As can be observed in Figure S3c,d (Supporting Information), the CeO_2_ particles are about 40 nm in size without the addition of nickel salt and cobalt salts, and the diameter of the CNTs is around 25 nm. The composite of CeO_2_ particles can also increase the surface area of the composite material, thereby the active sites can be increased, which is consistent with the results of the Brunauer–Emmett–Teller (BET) experiment. This kind of microstructured catalyst can provide enough catalytic surface to enlarge the area of contact between the cathode material and the electrolyte as well as the open space to facilitate the transfer of oxygen or lithium ions, thus providing more active sites for OER and ORR reactions, and sufficient specific surface district for the storage of discharge products. In addition, the porous structure of the nanotubes greatly promotes barrier‐free oxygen transmission and electrolyte penetration, and helps establish multi‐dimensional charge diffusion paths.^[^ ^54^ ^]^ The energy‐dispersive X‐ray spectroscopy diagram of Figure S4c–f in the Supporting Information shows the uniform distribution of Ni, Co, and Ce, indicating the successful recombination of Ce, which is also in accord with the results of Figure 1b. The atomic ratio of Ni to Co is about 1:2 (Table S1, Supporting Information), which is close to the original feed ratio, and the Ce content is about 18.7%. The above evidence demonstrates that after CeO_2_ is deposited, CeO_2_ nanoparticles are well coated on the surfaces of NiCo_2_O_4_ nanotubes, forming a typical hierarchical composite structure. It is noteworthy that although the surface roughness of NiCo_2_O_4_@CeO_2_ increases obviously compared with a single NiCo_2_O_4_ microsphere, the open voids are still able to be well preserved without being totally blocked, and the porous structure is retained even after the CeO_2_ particles are wrapped. The chemical composition and bonding state of NiCo_2_O_4_@CeO_2_ nanocomposites were deeper analyzed by X‐ray photoelectron spectroscopy (XPS), which was shown in Figure S5, Supporting Information.

**Figure 2:**
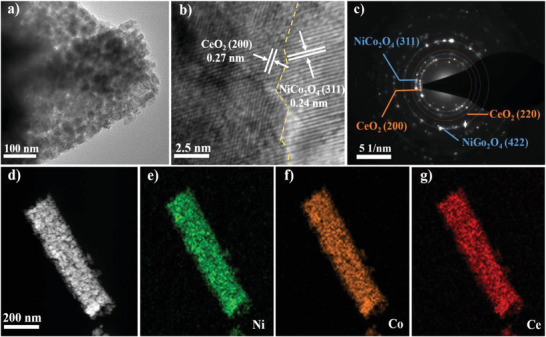
a) TEM image of NiCo_2_O_4_@CeO_2_ nanotubes. b) HRTEM image of NiCo_2_O_4_@CeO_2_ composite nanostructure. c) Electron diffraction pattern of NiCo_2_O_4_@CeO_2_. d–g) Elemental mapping images of mixed, Ni, Co, Ce of the NiCo_2_O_4_@CeO_2_ nanotube.

The more specific microstructure and morphology of NiCo_2_O_4_@CeO_2_ were studied via transmission electron microscope (TEM). **Figure** **2**a reveals the diameter of the nanotubes that make up NiCo_2_O_4_@CeO_2_ urchin‐like microspheres is about 200 nm, which is particularly consistent with the SEM image situated in the inset of Figure 1c. It is also found that the nanotubes are composed of self‐assembly of nanoparticles and they have a well‐constructed vesicular structure. The high‐resolution TEM (HRTEM) image in Figure 2b clearly shows a distinct heterogenous interface, with a lattice spacing of about 0.24 and 0.27 nm, which are in keeping with the (311) plane of NiCo_2_O_4_ and (200) plane of CeO_2_, respectively. This is in line with the results of the XRD pattern. The relevant selected area electron diffraction pattern (Figure 2c) shows clearly defined rings, indexed as (311) and (422) crystal planes of NiCo_2_O_4_ mixed with (200) and (220) crystal plane of CeO_2_. This indicates the polycrystalline nature of NiCo_2_O_4_@CeO_2_ urchin‐like microspheres, and further proves the successful preparation of NiCo_2_O_4_@CeO_2_ composites. In addition, the homologous energy dispersive X‐ray (EDX) element mapping image of a single nanorod (Figure 2d–g) reveals the uniform distribution of Ni, Co, and Ce elements, indicating that Ce has been successfully incorporated into the composite material. On the other hand, the type IV behavior with the existence of a hysteresis loop was determined by the typical nitrogen adsorption–desorption isotherm characteristics, as displayed in Figure S6 (Supporting Information).

### 2.2 Electrochemical Performances

**Figure 3:**
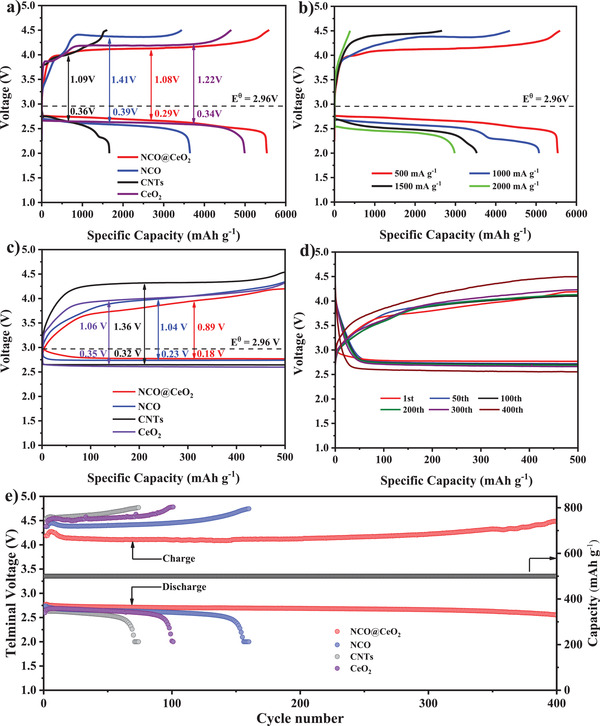
a) The initial discharge/charge curves of NiCo_2_O_4_@CeO_2_, NiCo_2_O_4_, CeO_2_, and pure CNTs electrodes at an ampere density of 500 mA g^−1^. b) The discharge/charge curves of NiCo_2_O_4_@CeO_2_ electrodes with different ampere densities. c) The initial discharge/charge curves of NiCo_2_O_4_@CeO_2_, NiCo_2_O_4_, CeO_2_, and CNTs electrodes with a cut‐off capacity of 500 mA h g^−1^ at an ampere density of 500 mA g^−1^. d) Typical discharge/charge curves of NiCo_2_O_4_@CeO_2_ electrode with different cycles at 500 mA g^−1^ under a limited capacity of 500 mA h g ^−1^. e) Cycling properties of NiCo_2_O_4_@CeO_2_, NiCo_2_O_4_, CeO_2_, and CNTs electrodes with a limited capacity of 500 mA h g^−1^ at 500 mA g^−1^.

In order to study the catalytic behavior of this composite material, NiCo_2_O_4_@CeO_2_ was employed as the cathode active substance of nonaqueous LOBs for charge and discharge tests. For comparison, NiCo_2_O_4_, CeO_2_, and pure CNTs‐based cathode materials were also evaluated under similar conditions. **Figure** **3**a shows the premier discharge/charge curves of NiCo_2_O_4_@CeO_2_, NiCo_2_O_4_, CeO_2_, and pure CNTs electrodes under the voltage window within 2.0–4.5 V at an ampere density of 500 mA g^−1^. Note that the charge and discharge capacity of pure CNTs‐based cathodes can be negligible, which indicates that the capacity contribution mainly comes from the active material. NiCo_2_O_4_@CeO_2_ catalyst presents a full charge and discharge capacity of 5537/5586 mA h g^−1^ with a relevant Coulombic efficiency of 99.1%, higher than 4655/4990 mA h g^−1^ of CeO_2_ with 93.2% and 3439/3646 mA h g^−1^ of NiCo_2_O_4_ with 94.3%, which confirms that NiCo_2_O_4_@CeO_2_ composite material can improve the Coulombic efficiency. More significantly, the NiCo_2_O_4_@CeO_2_ electrode shows the lowest charge (0.89 V) and discharge (0.18 V) medium capacity potential, which is defined as the potential at half‐capacity, leading to a pretty less polarization of only 1.07 V. As for NiCo_2_O_4_@CeO_2_ cathode, the charging and discharging processes are considerably stable with a fairly smooth voltage plateau in a broad capacity scope, which means that the formation and decomposition of Li_2_O_2_ continue to occur at a nearly steady reaction rate.^[^ ^55^ ^]^ These results illustrate that the composite material can significantly increase the specific capacity and reduce the overpotential of the LOBs, indicating that NiCo_2_O_4_@CeO_2_ has high electrocatalytic activity and effective reaction kinetics.

Figure 3b records the rate performance of the NiCo_2_O_4_@CeO_2_ composite electrode in a 2.0–4.5 V voltage window when the ampere densities increase from 500, 1000, 1500 to 2000 mA g^−1^. It could be seen that the NiCo_2_O_4_@CeO_2_ electrode exhibits discharge capacities of 5586 mA h g^−1^ at 500 mA g^−1^ and 6120 mA h g^−1^ at 1000 mA g^−1^. Even at the high current density of 2000 mA g^−1^, the NiCo_2_O_4_@CeO_2_ electrode still has a discharge capacity of 2979 mA h g^−1^. Interestingly, with a moderate ampere density of 1000 mA g^−1^, the capacity retention rate of NiCo_2_O_4_@CeO_2_ electrode is up to 85.7%, showing excellent rate capability. Although the capacity and efficiency, as well as the overpotential of the composite material, deteriorate with the increase of current density due to the growing internal transfer impedance (*R*_ct_) of the whole LOBs,^[^ ^56^ ^]^ they still show excellent performance among similar materials. This could be imputed to the unique recombination interface of NiCo_2_O_4_@CeO_2_ electrode that enhances electronic interaction, and the existence of vacancies can promote rapid electron transfer, leading to the reversible formation and decomposition of Li_2_O_2_.^[^ ^57^ ^]^ Figure 3c displays the initial discharge/charge profiles of the four samples with a limited capacity of 500 mA h g^−1^ with an ampere density of 500 mA g^−1^. The NiCo_2_O_4_@CeO_2_ electrode shows a smaller polarization than the NiCo_2_O_4_ cathode, CeO_2_ cathode, and pure CNTs cathode, with discharge/charge overpotentials of 0.89/0.18, 1.04/0.23, 1.22/0.50, and 1.36/0.32 V, respectively. NiCo_2_O_4_@CeO_2_ electrode presents better ORR and OER catalytic performance because the overpotential is the lowest (1.07 V), indicating that this active material can effectively reduce the overpotential.

In order to ensure the reversibility of oxygen reduction/evolution, we tested the LOBs with a limited charge/discharge depth, for which the cycle properties of the battery were measured when the ampere density is 500 mA g^−1^ and the cut‐off capacity for the LOBs is limited to 500 mA h g^−1^. The typical discharge/charge curves corresponding to different cycles of NiCo_2_O_4_@CeO_2_ electrode (Figure 3d) suggest that the termination charge voltage hardly increased during the first 300 cycles and no decline is observed, which is better than that of NiCo_2_O_4_, CeO_2_, and pure CNTs (Figure S7a–c, Supporting Information) during cycling. Figure S8a in the Supporting Information tests the charge–discharge rate performance of NiCo_2_O_4_@CeO_2_ electrode at different current densities from 200 to 1000 mA g^−1^ when the constant limited capacity is 500 mA h g^−1^. As shown in Figure 3e and Figure S8b in the Supporting Information, the charging voltage of all electrodes abruptly increases during the first five cycles, and then gradually stabilizes. The possible reason is that the reaction was not complete at the beginning, and Li_2_O_2_ was not decomposed in time. Under the same conditions, the termination charge voltages of pure CNTs, CeO_2_, and NiCo_2_O_4_ cathodes exceed 4.5 V in a short time, and then the terminal discharge voltage begins to drop sharply, showing limited cycle numbers of 70, 100, and 155 cycles, respectively. In sharp contrast to NiCo_2_O_4_ and CeO_2_ electrodes, NiCo_2_O_4_@CeO_2_ electrode can achieve 400 times longer cycle life in 2.0–4.5 V, and shows impressive cycle performance. From the above results, it can be concluded that the NiCo_2_O_4_@CeO_2_ composite material as an effective bi‐functional catalyst shows a huge advantage in nonaqueous LOBs. The synergistic effect of NiCo_2_O_4_ and CeO_2_ enhances its catalytic activity, and has excellent charge and discharge capabilities as well as cycle stability. The catalytic activity and redox mechanism of NiCo_2_O_4_@CeO_2_, NiCo_2_O_4_, CeO_2_, and CNTs were studied by cyclic voltammetry (CV) within a voltage window of 2.0–4.5 V relative to Li/Li ^+^ at a scanning speed of 0.1 mV s ^−1^ (Figure S9a, Supporting Information). To demonstrate the excellent kinetic performance of the composite electrode and greatly enhance the catalytic performance of the LOBs, electrochemical impedance spectroscopy (EIS) tests were accomplished on the different states of the electrode in the frequency scope of 100 kHz to 0.01 Hz (Figure S9b,c, Supporting Information). Besides, to understand the final product on the electrode surface, the cell was disassembled in the glove box and the electrode was washed three times with ethylene glycol dimethyl ether. The morphology after charging and discharging is shown in Figure S10 (Supporting Information). Table S2 in the Supporting Information also shows the comparison of the catalyst in this work with other related NiCo_2_O_4_ or CeO_2_ cathode materials for LOBs. These results indicate that the interaction between NiCo_2_O_4_ and CeO_2_ may lead to excellent electronic/ion conductivity and improve catalytic performance. The formed nanotubes are open and porous, which not only facilitates the transmission of lithium ions and oxygen, but also greatly enhances the ion diffusion ability.

### 2.3 Density Functional Theory Calculations

**Figure 4:**
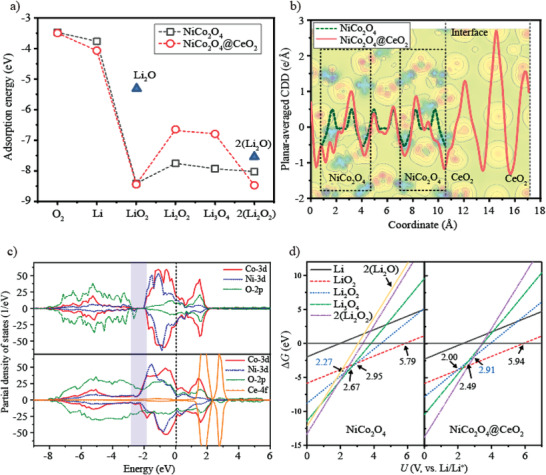
a) Adsorption energy (*E*_ads_) of the ORR intermediates (O_2_, Li, LiO_2_, Li_2_O, Li_2_O_2_, Li_3_O_4_, 2(Li_2_O), and 2(Li_2_O_2_) on the NiCo_2_O_4_ surface and NiCo_2_O_4_@CeO_2_ surface. b) Planar‐averaged CDD of NiCo_2_O_4_ surface and NiCo_2_O_4_@CeO_2_ surface. The red solid line represents the value of NiCo_2_O_4_@CeO_2_ surface, and the green dotted line is that of NiCo_2_O_4_ surface for comparison. c) PDOS of the clean NiCo_2_O_4_ and NiCo_2_O_4_@CeO_2_. d) Potential‐dependent profiles of intermediate discharge adsorption to the surface of NiCo_2_O_4_ surface and NiCo_2_O_4_@CeO_2_ surface.

In order to gain deep insight into the effect of CeO_2_ on the catalytic properties of NiCo_2_O_4_ for the OER at the atomic level, we implemented density functional theory (DFT) calculations to investigate the ORR and OER processes from both the thermodynamic and electrochemical perspectives. The computing approach has a detailed description in the Experimental Section. Primarily, the adsorption energy (*E*_ads_) of the ORR intermediate was calculated to determine the possible adsorption states at the same surface site, as shown in **Figure** **4**a. As the adsorption of O_2_ and Li on the surface of NiCo_2_O_4_ and NiCo_2_O_4_@CeO_2_, the value of *E*_ads_ gradually decreases, especially for the LiO_2_ molecular, of which the *E*_ads_ presents the maximal variance. It follows that the adsorption energy of the first lithiation step on these two surfaces is lower than that of O_2_, indicating the reduction of O_2_ on a clean surface of NiCo_2_O_4_ and NiCo_2_O_4_@CeO_2_ is easier than oxidation. As the adsorption continues, the NiCo_2_O_4_ and NiCo_2_O_4_@CeO_2_ surfaces show significantly different adsorption characteristics. Around the interface of NiCo_2_O_4_@CeO_2_, the adsorption energies of the intermediate state Li_2_O_2_ and Li_3_O_4_ become relatively weak, while a large decrease in variance occurs at the stage of 2(Li_2_O_2_). This observation is not notable in NiCo_2_O_4_, suggesting that NiCo_2_O_4_@CeO_2_ is more likely to be covered by the Li_2_O_2_ aggregates than NiCo_2_O_4_. On the other hand, the weak *E*_ads_ of Li_2_O and 2(Li_2_O) suggests that the two surfaces conduct catalytic processes through the favorable 2e^−^ pathway, thus confirming the experimental speculation. It can be inferred that the most significant change for NiCo_2_O_4_@CeO_2_ occurs in the adsorption of LiO_2_ to 2(Li_2_O_2_) molecules, because it largely determines the charge and discharge voltage, thereby affecting the catalytic efficiency.

Then, we performed planar‐average charge density difference (CDD) analysis (Figure 4b) to obtain the charge‐transfer properties near the interface of NiCo_2_O_4_@CeO_2_. For comparison, the CDD of NiCo_2_O_4_ under the same spatial phase and scale is presented by the green dashed line. It is observed that excess electrons are obtained by Ce and O atoms of CeO_2_, forming an electron gas near the interface. As a result, the atoms of the NiCo_2_O_4_ termination lost excess electrons at the interface. The charge transfer from NiCo_2_O_4_ to CeO_2_ also leads to a drastic charge fluctuation in NiCo_2_O_4_, which exists in the range of at least four atomic layers. Figure 4c shows the partial density of states (PDOS) of NiCo_2_O_4_ and NiCo_2_O_4_@CeO_2_, respectively, which reveals that these two systems have metallic properties. Except that the PDOS of NiCo_2_O_4_@CeO_2_ is increased at the Fermi level compared with NiCo_2_O_4_, the remarkable difference between them is that the electronic orbital overlap for the former is more significant, which can facilitate the charge transfer capability during the electrochemical reaction.

Before analyzing the electrochemical reaction in these two systems, we constructed a phase diagram to quantify the stability of the ORR product, as shown in Figure 4d. By comparison, the Li_2_O_2_ nucleation is initiated when the electrode potential drops to 2.95 V, while on the NiCo_2_O_4_@CeO_2_ surface, Li_2_O_2_ nucleates below 2.00 V. The next ORR step on NiCo_2_O_4_ and NiCo_2_O_4_@CeO_2_, which is the formation of Li_3_O_4_ and 2(Li_2_O_2_), occurs below 2.95 and 2.67 V for NiCo_2_O_4_, while 2.49 and 2.91 V for NiCo_2_O_4_@CeO_2_. The higher Δ*G* of 2(Li_2_O) also confirms that NiCo_2_O_4_ and NiCo_2_O_4_@CeO_2_ adsorb middle discharge products via the thermodynamically favorable 2e^−^ route, resulting in the formation of 2(Li_2_O_2_). In contrast to the discharge course, which involves the middle‐products adsorption of Li*_p_* O*_q_*, the charging reaction occurs straightly through the decomposition of Li_2_O_2_ at the electrolyte/Li_2_O_2_ interface during the OER process. On the base of Figure 4d, beyond 2.27 and 2.91 V, which are the intersections of the 2(Li_2_O_2_) and Li_2_O_2_ lines for NiCo_2_O_4_ and NiCo_2_O_4_@CeO_2_, respectively, 2(Li_2_O_2_) dissociates to 2(Li_2_O_2_), 2Li, and O_2_. These threshold potentials correspond to the minimum charge potentials (see discussion below). The lower charge potential of NiCo_2_O_4_@CeO_2_, when compared to NiCo_2_O_4_ strongly, suggests that Li_2_O_2_ separates more easily.

**Figure 5:**
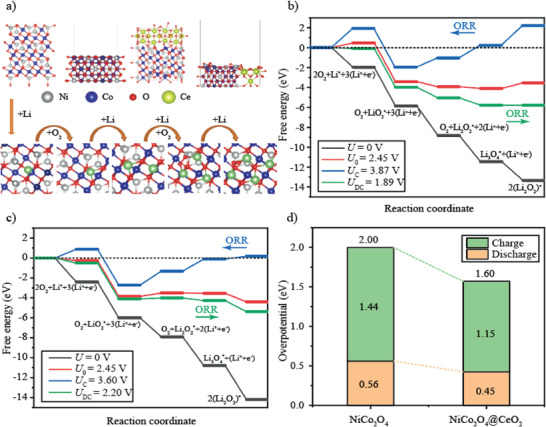
a) Top and side views of the ORR mechanism for the O_2_ reduction near the NiCo_2_O_4_ or NiCo_2_O_4_@CeO_2_ surface. Free energy diagrams for the charge and discharge process on the b) NiCo_2_O_4_ surface and c) NiCo_2_O_4_@CeO_2_ surface. The asterisk labels the adsorbed states of the molecules. d) Computed charge overpotentials (*η*_C_), discharge overpotentials (*η*_DC_), and the aggregated overpotentials (*η*_Total_) on the NiCo_2_O_4_ and NiCo_2_O_4_@CeO_2_ surfaces. The green and orange bars represent *η*_C_ and *η*_DC_, respectively, and the *η*_Total_ is displayed above the stacked bars.

**Figure** **5**a–c shows the illustration of every reaction route consisting of the five foundational reaction steps, as well as the corresponding free energy charts traced with the OER and the ORR routes with the most stable adsorption configurations of the ORR middle‐products on the NiCo_2_O_4_ and NiCo_2_O_4_@CeO_2_ surfaces, respectively. To demonstrate the electrocatalytic activity on every surface, discharge potentials (*U*_DC_) and the charge potentials (*U*_C_) were evaluated as the maximum and minimum voltages, respectively, causing every route to maintain declivity. It can be concluded that the NiCo_2_O_4_@CeO_2_ surface outperforms the NiCo_2_O_4_ surface. In this case, the *U*_C_ of the NiCo_2_O_4_@CeO_2_ surface is noticeably better, with a very low potential of 3.60 V than that of the NiCo_2_O_4_ surface (3.87 V). On the other hand, with the higher voltage of 2.20 V, the promotion of the *U*_DC_ on the NiCo_2_O_4_@CeO_2_ surface appears more prominent.

To help clarify how much this promotion in the electrocatalytic activity is corresponding to the electro‐catalytic efficiency of NiCo_2_O_4_@CeO_2_ surfaces, the overpotentials are evaluated as shown in Figure 5d. The following formula is used to calculate the overpotential during the first charge and discharge process, 1) *η*_C_ = *U*_C_ – *U*_0_, 2) *η*_DC_ = *U*_0_ − *U*_DC_. From the results in Figure 5d, it can be seen that when NiCo_2_O_4_ is used as the cathode catalyst of LOBs, the total overpotential for the first charge and discharge is 2.00 V, and when the CeO_2_ is doped into NiCo_2_O_4_, the total overpotential drops to 1.60 V. CeO_2_ doping of NiCo_2_O_4_ reduces the overpotential of the OER and ORR processes during the charging and discharging of the lithium–air battery, and the overpotential reduction (0.29 V) of the OER process is greater than that of the ORR process (0.11 V). Evidently, the NiCo_2_O_4_@CeO_2_ acts as a double‐way catalyst that plays a role in both the charge and discharge process, which is consistent with the experimental detection.

## 3 Conclusions

In summary, we synthesized a new type of porous urchin‐like NiCo_2_O_4_@CeO_2_ composite microspheres as a bi‐functional catalytic cathode for OER and ORR in LOBs. A number of CeO_2_ nanoparticles are tightly adsorbed on NiCo_2_O_4_ nanotubes, forming a rich interface. DFT calculations show that there is a strong chemical interaction between NiCo_2_O_4_ and CeO_2_, which stimulates the electronic conductivity of the composite material. When tested in the LOBs, the NiCo_2_O_4_@CeO_2_ electrode shows a relatively low overpotential of 1.07 V, and a highly long life of 400 cycles under a fixed capacity of 500 mA h g^−1^ at 500 mA g^−1^. The noteworthy improvement in the electrocatalytic properties of ORR/OER is attributed to the open structure of the composite material and the synergistic effect of the customized catalytic material. The unique porous structure can establish rapid oxygen and electron transport in the cathode and provide enough space for Li_2_O_2_ storage. These characteristics enable NiCo_2_O_4_@CeO_2_ to exhibit significantly improved OER/ORR electrocatalytic activity compared with the simplex components of NiCo_2_O_4_ and CeO_2_. Additionally, with the help of DFT calculations, we clarified the 2e^−^ pathway of NiCo_2_O_4_@CeO_2_ during ORR/OER, as well as the enhancement mechanism of electrochemical performance as a cathode catalyst for LOBs from the perspective of electronic structure and reaction kinetics. This work not only demonstrates a highly efficient oxygen electrocatalyst, but also provides a strategy to improve electrocatalytic performance for LOBs through interface/surface engineering.

## 4 Experimental Section

### Material Preparation

At room temperature, NiCl_2_·6H_2_O (0.297 g), CoCl_2_·6H_2_O (0.595 g), cerium acetate (0.181 g), and urea (0.27 g) were deliquescent in ultrapure water (25 mL), and the liquor was sonicated for 0.5 h. Then it was removed to a 50 mL stainless steel autoclave lined with polytetrafluoroethylene and heated to 120 °C for 8 h. After cooling to room temperature, the product was obtained by centrifugation, washed three times with deionized water and ethanol, respectively, and then dried overnight under 80 °C vacuum. Finally, the collected product was calcined at 375 °C with a temperature rate of 1 °C min ^−1^ for 2 h to obtain urchin‐like NiCo_2_O_4_@CeO_2_ microspheres in the air. For comparison, NiCo_2_O_4_ microspheres and CeO_2_ nanoparticles were also prepared by the same procedure only by adding the corresponding metal salt.

### Materials Characterization

The powder XRD patterns were measured on Bruker‐Axs D8 X‐ray diffractometer using Cu K_*α*_ radiation at 40 kV and 40 mA. The XPS information was performed by a Thermo Scientific K‐Alpha instrument with an Al K_*α*_ source. The surface morphological characteristics of the products were analyzed by using SEM (Hitachi SU‐70). TECNAI F‐30 TEM operating at 300 kV was acquired to observe the microstructure of the samples. The BET specific surface area and Barrett–Joyner–Halenda pore volume of materials were measured using 3H‐2000PM2 analyzer.

### Electrochemical Measurements

2032‐type coin cells with 19 small holes on the cathode side were packed in an Ar‐filled glove‐box (the content of moisture and oxygen <0.1 ppm). Each cell was made up of a fresh Li metal plate as the anode, a glass fiber separator (Whatman, GF/D), 1 m LiTFSI in tetraethylene glycol dimethyl ether as the electrolyte, a piece of carbon paper coated with catalyst as cathode, and a porous nickel foam as O_2_ diffusion layer. 40 wt% catalyst, 40 wt% mCNT, and 20 wt% polyvinylidene fluoride were evenly mixed with the limited *N*‐methyl pyrrolidone solution, which was painted on 12 mm carbon paper homogenously using paintbrushes. The samples were dried in a vacuum under 80 °C for 12 h, and the mass loading on the cathode was about 0.3–0.5 mg. After the assembled battery was put in the glove box for 24 h, it was transferred to a bottle full of oxygen, and the NEWARE BTS battery charging system was used at room temperature to perform constant current charge and discharge test in the voltage range of 2.0–4.8 V, and calculate current density and specific capacity by weight according to the cathode catalyst. CV was performed on the CHI660D electrochemical workstation (CH Instrument Co., Ltd., Shanghai, China), with a scan rate of 0.1 mV s^−1^, within a voltage window of 2.0–4.8 V (vs Li/Li^+^). EIS was performed on the same electrochemical workstation, and the frequency range was 100 KHz to 0.1 Hz.

### Calculation Methods

The DFT calculations were implemented in the Vienna ab initio simulation package (VASP) with a plane‐wave basis set and a projector‐augmented wave method.^[^ ^58^, ^59^ ^]^ The electron–core interactions were represented by generalized gradient approximation in the parametrization of the Perdew–Burke–Ernzerhof (PBE) pseudopotential,^[^ ^60^ ^]^ and the cutoff of the plane‐wave kinetic energy was 400 eV. To solve the underestimation problem of the Ce 4f states by the standard PBE, the DFT+U method was employed and the on‐site Coulomb interaction correction with an effective *U* value of 5.0 eV was applied in the highly localized Ce 4f states.^[^ ^61^ ^]^ The van der Waals interaction was described by using the empirical correction in Grimme's scheme, i.e., DFT + D3.^[^ ^62^ ^]^ Spin‐polarized calculations were applied in all the cases.

NiCo_2_O_4_ and NiCo_2_O_4_@CeO_2_ were modeled to identify the catalytic activity of the interface formed by NiCo_2_O_4_ and CeO_2_, which was helpful to understand the catalytic mechanism. For convenience, NiCo_2_O_4_@CeO_2_ was constructed containing two nearly symmetric NiCo_2_O_4_ (422)||CeO_2_(220) interfaces, whose rationality can be found in both the literature^[^ ^63^ ^]^ and the comparison with the experimental results. The vacuum space was greater than 15 Å, which was sufficient to prevent interaction between periodic images. The adsorption energies relevant to the possible stable configuration of the ORR intermediate products, i.e., (Li)^*^, (LiO_2_)^*^, (Li_2_O_2_)^*^, (Li_3_O_4_)^*^, and 2(Li_2_O_2_)^*^ were used to construct free energy diagrams. The Gibbs free energies of these molecules as a result of electrochemical adsorption reactions were calculated using the following expression

(1) $$\Delta G\;=\;\Delta E_{\mathrm{tot}}+\Delta E_{\mathrm{ZPE}}-T\Delta S\;$$

where Δ*E*_tot_ is the change in total energy obtained from DFT, Δ*E*_ZPE_ and *S* are the changes in zero‐point energy and entropy at standard conditions (*T* = 298 K and 0 V vs Li/Li^+^ reference electrode for O_2_ reduction).

The adsorption energies of Li*_p_* O*_q_* intermediates, for a given site, were defined as

(2) $$E_{\mathrm{ads}}=E_{\mathrm{surf}+Li_{p}O_{q}}\;-E_{\mathrm{surf}}-E_{Li_{p}O_{q}}$$

where *E*_surf_ is the surface energy, $E_{Li_{p}O_{q}}$ is the energy of molecular Li*_p_* O*_q_* species, and $E_{\mathrm{surf}+Li_{p}O_{q}}$ is the total system energy.

It should be noted that in this method, lower adsorption energy value, *E*_ads_, implied stronger binding between adsorbate molecules and the NiCo_2_O_4_ or NiCo_2_O_4_@CeO_2_ surface. The equilibrium potential for bulk Li_2_O_2(s)_ (Föppl structure) was calculated using the standard free energies of formation as follows

(3) $$U_{\mathrm{eq}}=\;-\frac{1}{ne}\left[\Delta G_{f}^{0}\left(Li_{2}O_{2\left(s\right)}\right)\right]$$

where the standard free energy of formation $\Delta G_{f}^{0}$ for the bulk Li_2_O_2(s)_ at 298 K was calculated as

(4) $$\Delta \;G_{f}^{0}=G_{Li_{x}O_{y\left(s\right)}}^{0}\;-xG_{Li_{\left(s\right)}}^{0}-\frac{y}{2}G_{O_{2\left(g\right)}}^{0}$$

The phonon contributions to entropy were contained in the *G* based on the harmonic approximation.

## Conflict of Interest

The authors declare no conflict of interest.

## Supporting information

Supporting Information

## Data Availability

The data that support the findings of this study are available from the corresponding author upon reasonable request.
