# Supplementary material for: Composite NiCo2O4@CeO2 Microsphere as Cathode Catalyst for High‐Performance Lithium–Oxygen Battery
Source: Adv Sci (Weinh). 2022 Apr 27;9(17):2200523. doi: 10.1002/advs.202200523 (PMC9189671; doi:10.1002/advs.202200523)
Supplement: Supplementary file 1 — Supporting Information [file ADVS-9-2200523-s001.pdf]

ADVANCED SCIENCE
Open Access

Supporting Information

for *Adv. Sci.*, DOI 10.1002/advs.202200523

Composite $NiCo_2O_4$@CeO_2 Microsphere as Cathode Catalyst for High-Performance Lithium–Oxygen Battery

Yuanhui Wu, Haoran Ding, Tianlun Yang, Yongji Xia, Hongfei Zheng, Qiulong Wei, Jiajia Han,, Dong-Liang Peng* and Guanghui Yue**

Supporting Information

Composite $NiCo_2O_4$@CeO_2 microsphere as cathode catalyst for high-performance lithium-oxygen battery

Yuanhui Wu, Haoran Ding, Tianlun Yang, Yongji Xia, Hongfei Zheng, Qiulong Wei, Jiajia Han, Dong-Liang Peng* and Guanghui Yue**

Y. Wu, H. Ding, T. Yang, Y. Xia, H. Zheng, Dr. Q. L. Wei, Dr. J. Han, Prof. D.L. Peng, Prof. G.H. Yue

State Key Lab of Physical Chemistry of Solid Surface, Fujian Key Laboratory of Materials Genome, Collaborative Innovation Center of Chemistry for Energy Materials, College of Materials, Xiamen University, Xiamen 361005, China.

Email: jiajiahan@xmu.edu.cn(J. Han); yuegh@xmu.edu.cn (G. Yue); dlpeng@xmu.edu.cn (D. Peng).

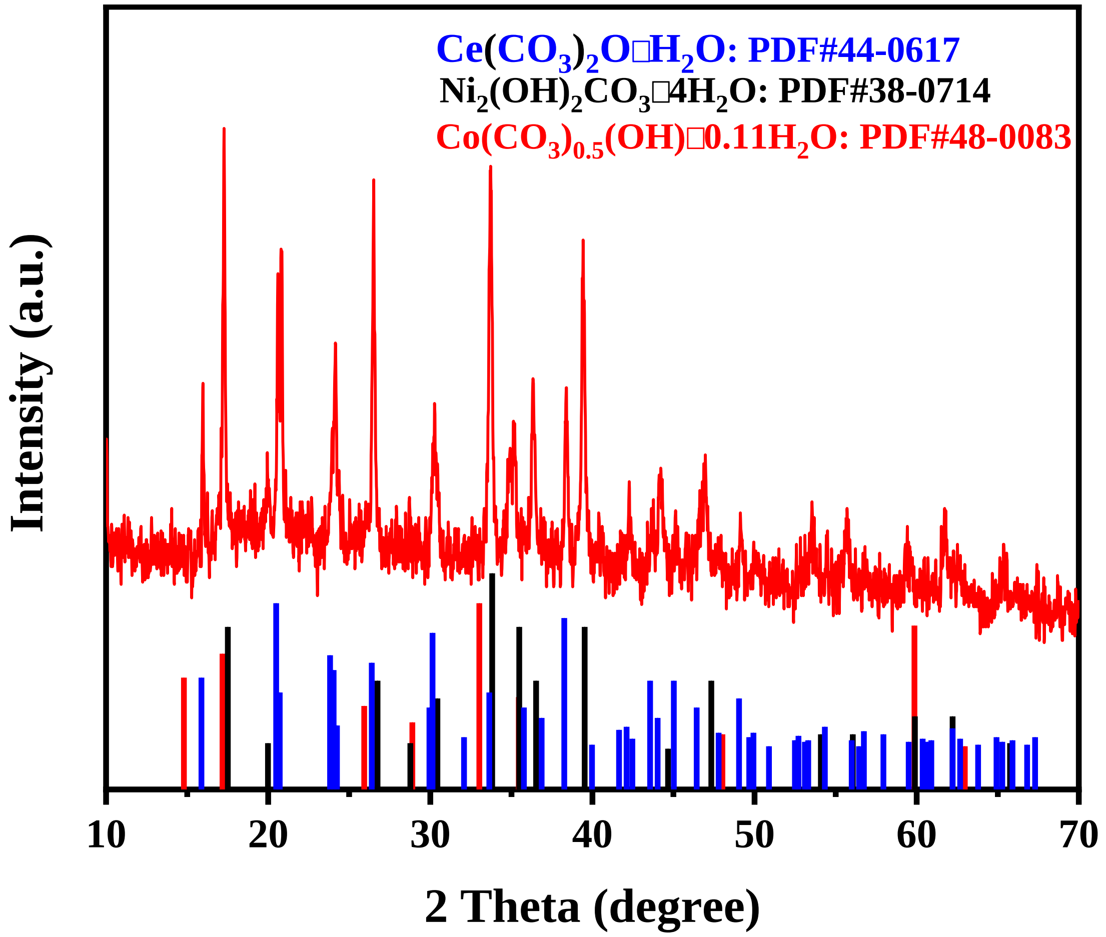

Figure S1. The XRD patterns of the precursor of $NiCo_2O_4@CeO_2$.

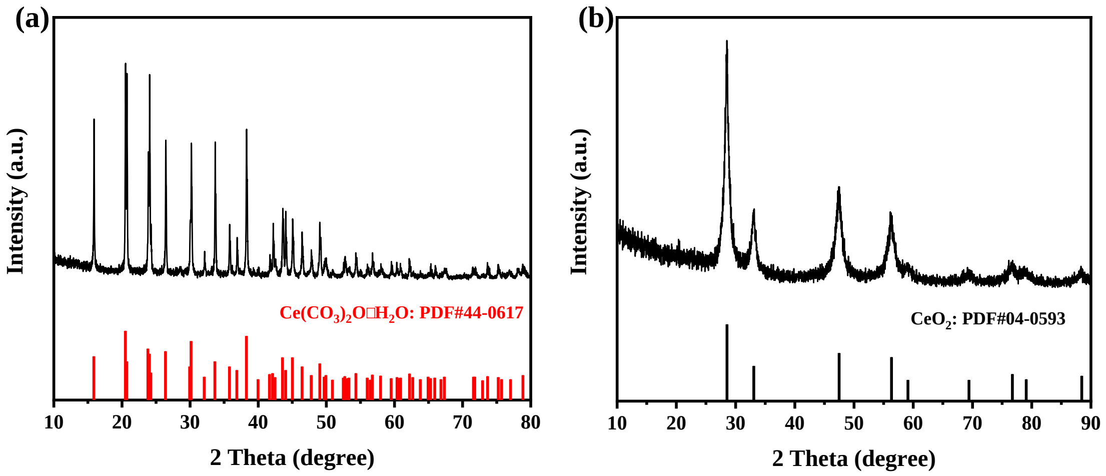

Figure S2. The XRD patterns of (a) the precursor of CeO_2 and (b) CeO_2.

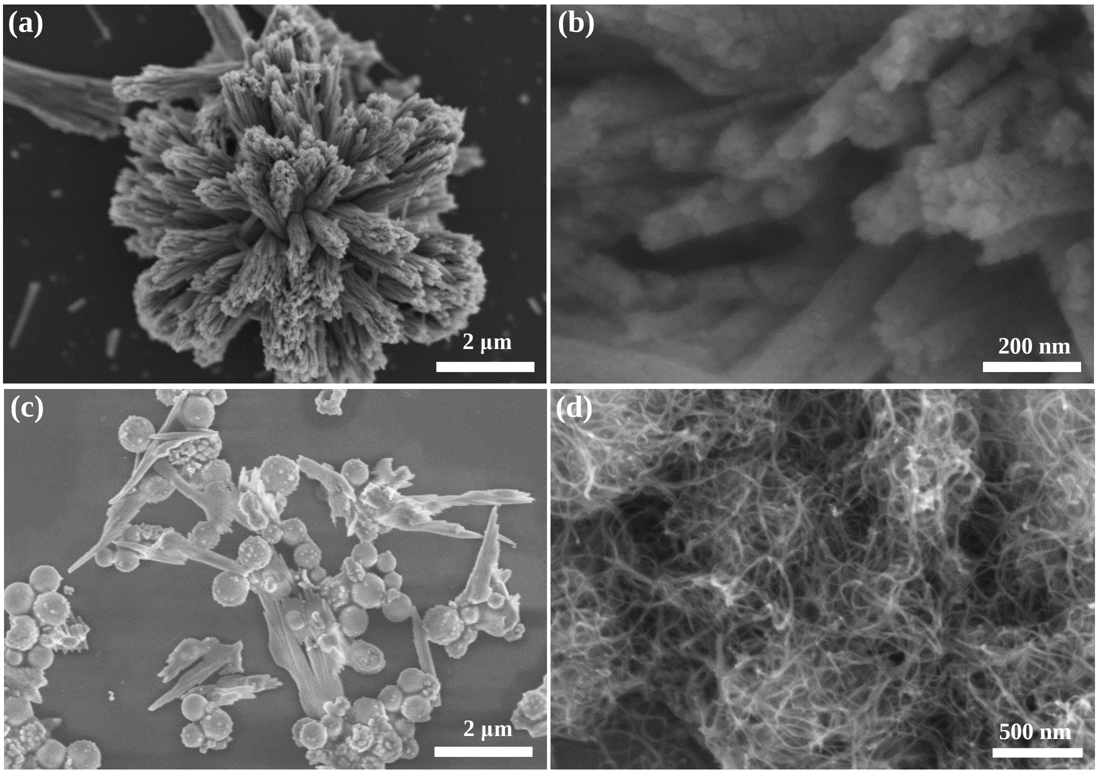

Figure S3. SEM images of (a) (b) $NiCo_2O_4$, (c) CeO_2 and (d) CNTs.

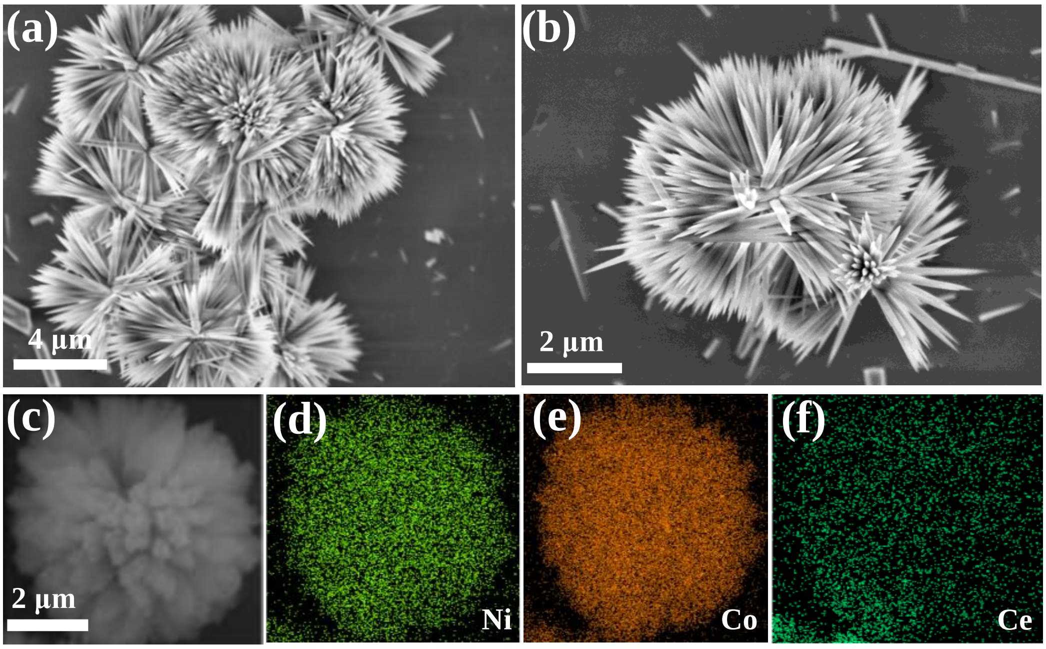

Figure S4. SEM images of (a) (b) $NiCo_2O_4@CeO_2$ precursor; (c) EDS results of $NiCo_2O_4@CeO_2$; element mapping of $NiCo_2O_4@CeO_2$: (d) Ni, (e) Co and (f) Ce.

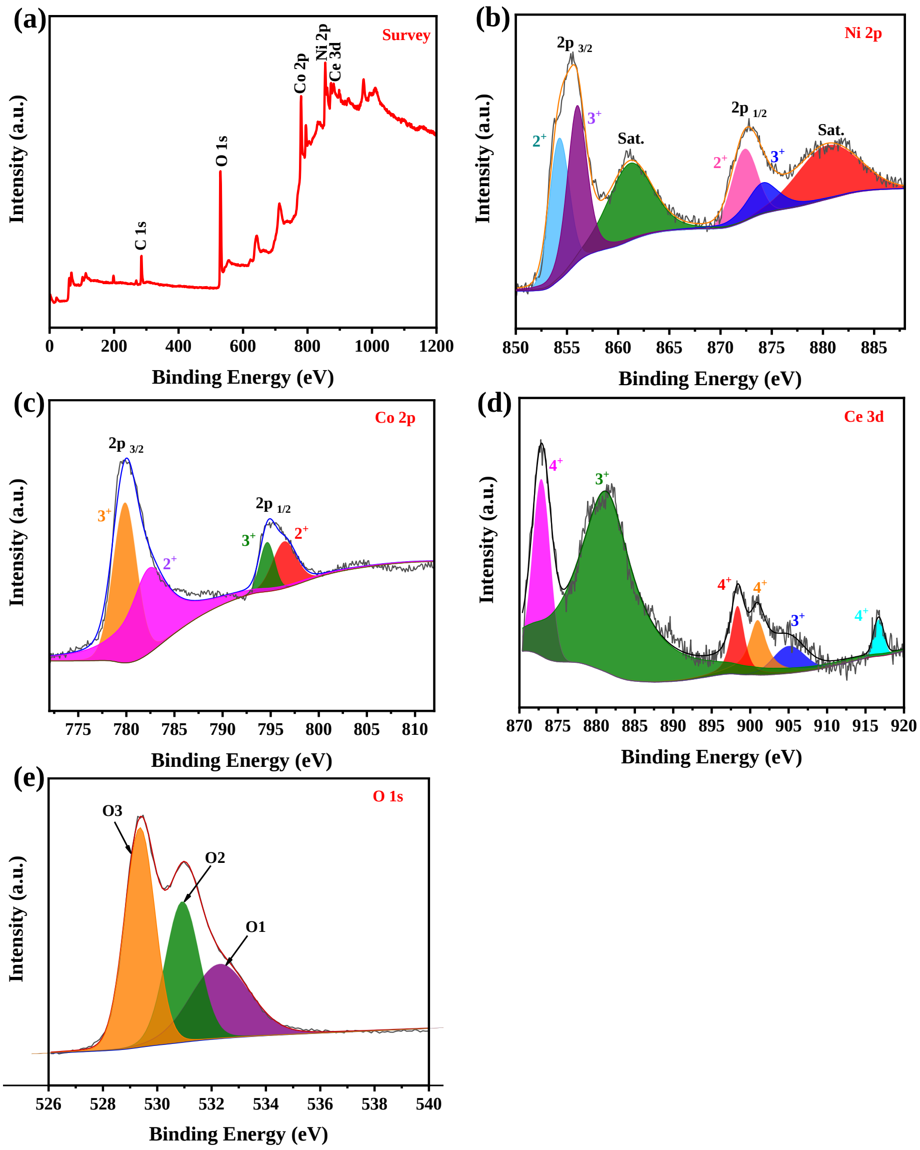

Figure S5. XPS spectra of $NiCo_2O_4@CeO_2$: (a) survey, (b) Ni 2p, (c)Co 2p, (d)Ce3d, and (e) O 1s.

As shown in the survey spectrum (**Figure S5**a), only Ni, Co, Ce and O were detected without other impurities. The XPS spectrum of high-resolution Ni2p based on Gaussian fitting is shown in **Figure S5**b, where two spin-orbit double peaks and two satellite peaks (identified as "Sat.") can be observed. The fitting peaks with binding energies of 854.2 and 872.4eV are Ni^{2+} signals, and those centered at 856.0 and 874.2 eV correspond to Ni^{3+} signals. The peaks at 861.3 and 880.7 eV can be

assigned to Ni satellite peaks.[1, 2] In the same way, Co2p can be fitted to two spin–orbit doublets (**Figure S5**c). The binding energies around 779.8 and 794.6 eV correspond to Co^{3+}, and the peaks around 782.6 and 796.5 eV can be interpreted as Co^{2+}.[3-5], The high-resolution Ce 3d spectrum in **Figure S5**d exhibits four peaks at 872.9, 898.0, 901.0 and 916.7 eV, which are consistent with Ce^{4+}, while the other two peaks at 881.1 and 905.2 eV indicate the presence of Ce^{3+} ions.[6, 7] The transition between Ce^{3+} and Ce^{4+} can promote the rapid electron transfer and ion diffusion from cerium oxide to the $NiCo_2O_4$ supported material.[8, 9] These results indicate that in $NiCo_2O_4$@CeO_2, Ni, Co and Ce are partially oxidized and reduced, respectively, to balance the formation of oxygen vacancies. From the O1s area in **Figure S5**e, the three contributions of oxygen can be clearly verified. The O1 peak at 529.3 eV is attributed to the typical metal-oxygen bond, the O2 peak at 530.9 eV corresponds to more defective sites with low oxygen coordination, which are usually observed in materials with small particles and a variety of surface substances (including hydroxyl groups, chemisorbed oxygen, or intrinsic substances on the spinel surface), and the O3 peak of 532.3 eV is usually related to the diversity of chemical adsorption and physical adsorption of water on or inside the sample.[10, 11] XPS results show that the $NiCo_2O_4$@CeO_2 composite contains mixed valence states Ni^{3+}, Ni^{2+}, Co^{3+}, Co^{2+}, Ce^{4+} and Ce^{3+}, which has excellent electrochemical reaction activity and provides abundant active sites for redox reactions.

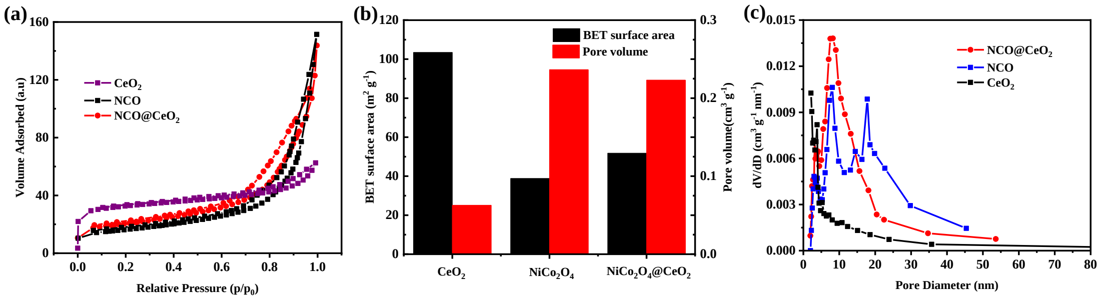

Figure S6. (a) The nitrogen adsorption–desorption isotherms; (b) The BET surface area and pore volume; (c) The pore diameter distribution of three samples.

The type IV behaviour with the existence of hysteresis loop was determined by the typical nitrogen adsorption–desorption isotherm characteristics, as shown in **Figure S6**a. It is indicated that a large amount of mesopores exist in $NiCo_2O_4$@CeO_2, which is consistent with the pore size distribution curve shown in **Figure S6**c. Abundant mesopores and macropores can provide a way for rapid mass transfer and accessible active sites for electrochemical reactions. BET was applied to investigate the specific surface area and pore volume of CeO_2, $NiCo_2O_4$ and $NiCo_2O_4$@CeO_2 (**Figure S6**b and **Figure S6**c). Compared with $NiCo_2O_4$ microspheres and CeO_2 nanoparticles, the low specific surface area and decreased pore volume of $NiCo_2O_4$@CeO_2 indicate that the pores on $NiCo_2O_4$ are mainly saturated with CeO_2 nanoparticles and the close contact between $NiCo_2O_4$ nanotubes and CeO_2 nanoparticles is essential to generate the synergy effect and electrochemical interaction between the two components.

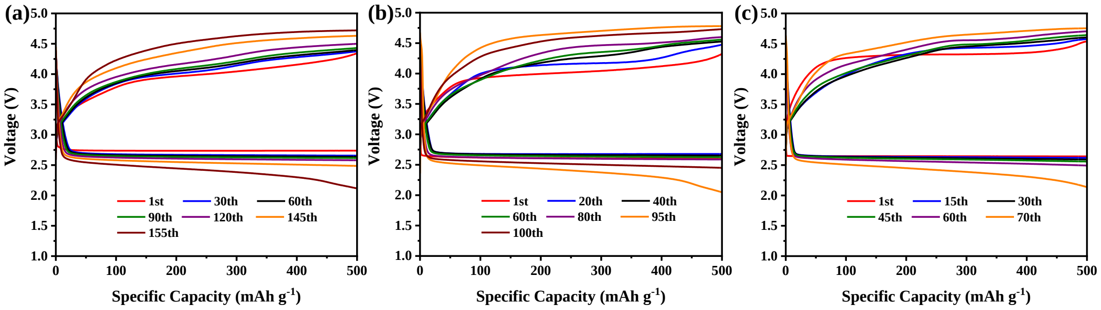

Figure S7. Cycle performance of (a) $NiCo_2O_4$, (b) CeO_2 and (c) CNTs.

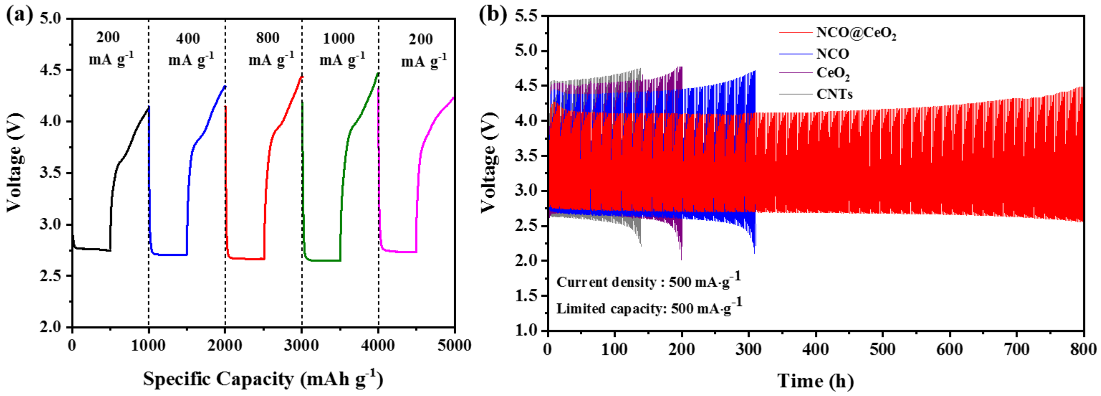

Figure S8. (a) The discharge–charge profiles of $NiCo_2O_4@CeO_2$ electrode at different current densities from 200 mA g^{-1} to 1000 mA g^{-1} with a constant cut-off capacity of 500 mA h g^{-1}; (b) Galvanostatic discharge/charge profiles of $NiCo_2O_4@CeO_2$, $NiCo_2O_4$, CeO_2 and CNTs electrodes at 500 mA g^{-1} with a fixed specific capacity of 500 mA h g^{-1}.

As we can see from **Figure S8**a, the discharge voltage is about 2.75 V at low current density (200 mA g^{-1}), and then at high current density (1000 mA g^{-1}), the discharge voltage slowly drops to 2.65 V. At the same time, the charging voltage changes steadily from 4.11 V to 4.46 V as the current density ranges from 200 mA g^{-1} to 1000 mA g^{-1}. The charge and discharge potentials show good reversibility when the current density returns to 200 mA g^{-1}. All this indicate that the $NiCo_2O_4@CeO_2$ electrode has excellent feasibility.

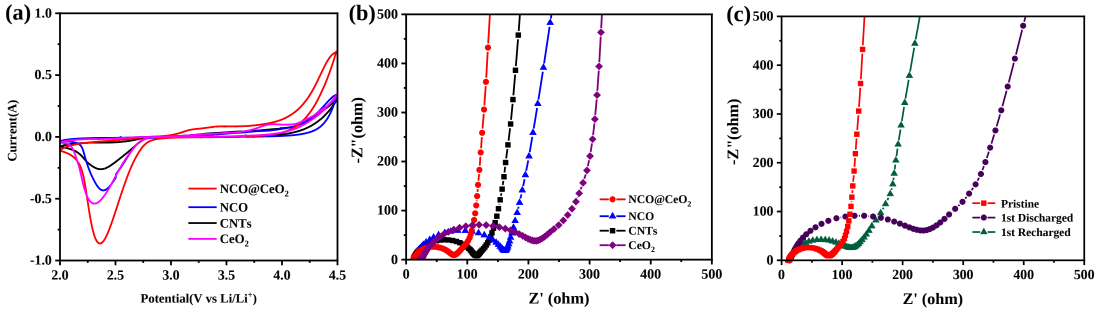

Figure S9. (a) CV curves of $NiCo_2O_4$@CeO_2, $NiCo_2O_4$, CeO_2 and CNTs electrodes; (b) Nyquist plots of $NiCo_2O_4$@CeO_2, $NiCo_2O_4$, CeO_2 and CNTs electrodes; (c) Nyquist plots of $NiCo_2O_4$@CeO_2 electrodes at different discharge/charge stages.

From **Figure S9**a, $NiCo_2O_4$@CeO_2, $NiCo_2O_4$, and CeO_2 electrodes all show an obvious single reduction peak. In particular, $NiCo_2O_4$@CeO_2 electrode has a higher cathodic peak potentials and considerable cathode current value compared with other electrodes. It is worth noting that a clear oxidation peak of $NiCo_2O_4$@CeO_2 electrode can be seen at 3.36 V, which is caused by the gradual delithiation and bulk oxidation process of Li_2O_2 ($Li_2O_2 \rightarrow O_2 + 2Li^+ + 2e^-$).[12, 13] For the CeO_2 electrodes, the anode peak appears at a higher 3.87 V. On the other hand, $NiCo_2O_4$@CeO_2 shows a higher peak current and a larger integration area compared with other samples, indicating that the composite electrode can produce more Li_2O_2 ($2Li^+ + O_2 + 2e^- \rightarrow Li_2O_2$) during the ORR process , and cause more Li_2O_2 reversible decomposition in the OER process, which is consistent with the initial charge-discharge curves in **Figure 3**a.[14, 15] During the scanning process of the electrode, the $NiCo_2O_4$@ CeO_2 composite cathode has a higher trigger potential for oxygen reduction and a lower trigger voltage for oxygen release, which is accordance with the voltage plateau observed in the constant current first discharge/charge curves in **Figure 3**c, showing excellent electrochemical

catalytic activity. These results indicate that the vacancies caused by CeO_2 can expose more defects, thereby providing abundant surface-active sites for O_2 adsorption and activation. It demonstrats the synergistic effect of $NiCo_2O_4$ and CeO_2 improves the catalytic kinetics of ORR and OER.

EIS is composed of a small semicircle in the high frequency area and an oblique straight line in the low frequency area. The small semicircle represents charge transfer resistance, and its diameter reflects the difficulty of charge transfer to a certain extent. The straight line represents the Warburg impedance.[16] The oblique line is related to the ion diffusion process in the electrode. From the Nyquist diagram (**Figure S9**b), it can be seen that the $NiCo_2O_4$@CeO_2 catalytic electrode has the smallest semicircle, that is, the charge transfer resistance R_{ct} is much lower than that of $NiCo_2O_4$, CeO_2 and CNTs. In addition, the Warburg slope is the largest, indicating that the $NiCo_2O_4$@CeO_2 composite electrode has high electronic conductivity and can be used for effective charge transfer at the electrode/electrolyte interface, which ensures electrochemical activity during cycling.[17] EIS tests of $NiCo_2O_4$@CeO_2 electrodes at different discharge/charge stages (**Figure S9**c) were also carried out to further determine the discharge and charge characteristics. It can be clearly seen that after the first discharge, a larger charge transfer resistance is observed compared to the fresh electrode. This is because the formation and accumulation of the insulating discharge product Li_2O_2 on the electrode can block the active sites to a large extent, preventing electron transfer and causing high resistivity. After recharging, the impedance of the $NiCo_2O_4$@CeO_2 electrode is not much different from the initial value, indicating that

the formation and decomposition of reaction products are reversible.

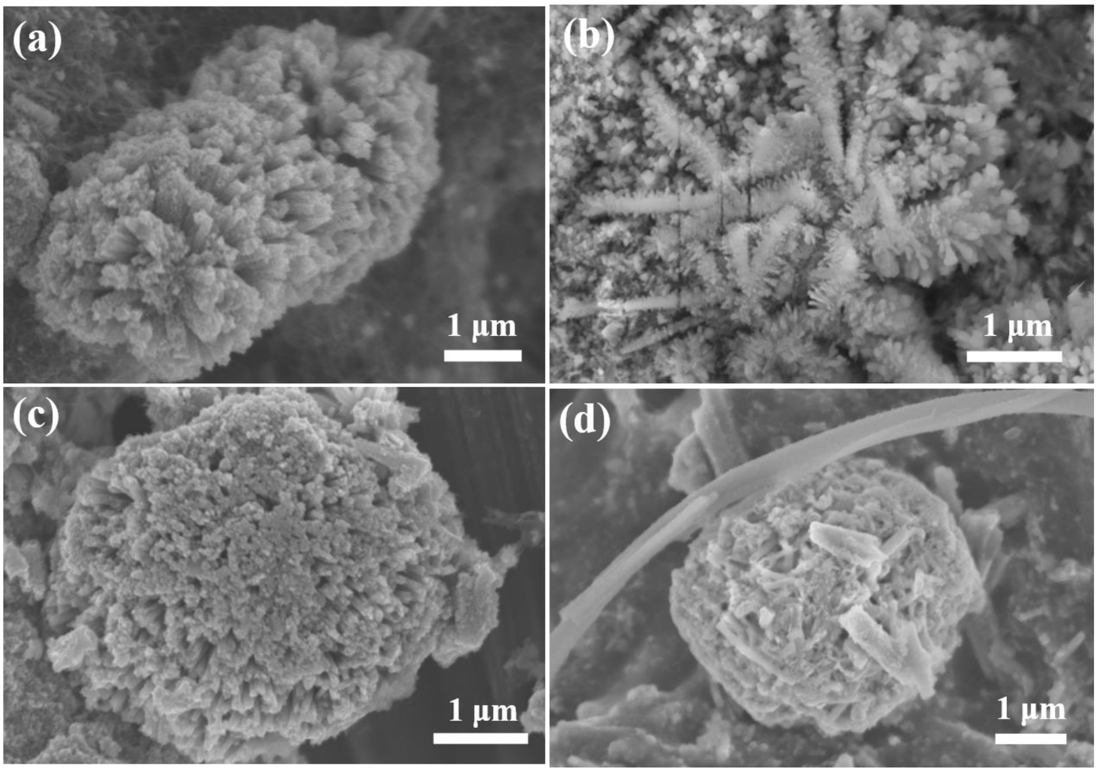

Figure S10. SEM images of $NiCo_2O_4$@CeO_2 electrodes at 500 mA g^{-1} with a limited capacity of 500 mA h g^{-1}: (a) initial electrodes; (b) first discharged electrodes; (c) first charged electrodes; (d) electrodes after 200 cycles.

In addition, we also further characterized the morphology of the $NiCo_2O_4$@CeO_2 catalyst electrode after charging and discharging process, while the current density was 500 mA g^{-1} and the limited capacity was 500 mA h g^{-1}. The original electrode is presented in **Figure S10**a. As shown in **Figure S10**b, granular Li_2O_2 is formed on the surface of the pole piece after the first discharge of $NiCo_2O_4$@CeO_2 electrode. After subsequent charging, the Li_2O_2 on the surface of the $NiCo_2O_4$@CeO_2 electrode completely disappeared and completely recovered to the initial state before (**Figure S10**c). **Figure S10**d shows that the pole piece still maintains the urchin-like morphology after 200 cycles, and the surface is slightly broken. These results indicate

that $NiCo_2O_4@CeO_2$ can significantly increase the OER performance, thereby promoting the decomposition of Li_2O_2.

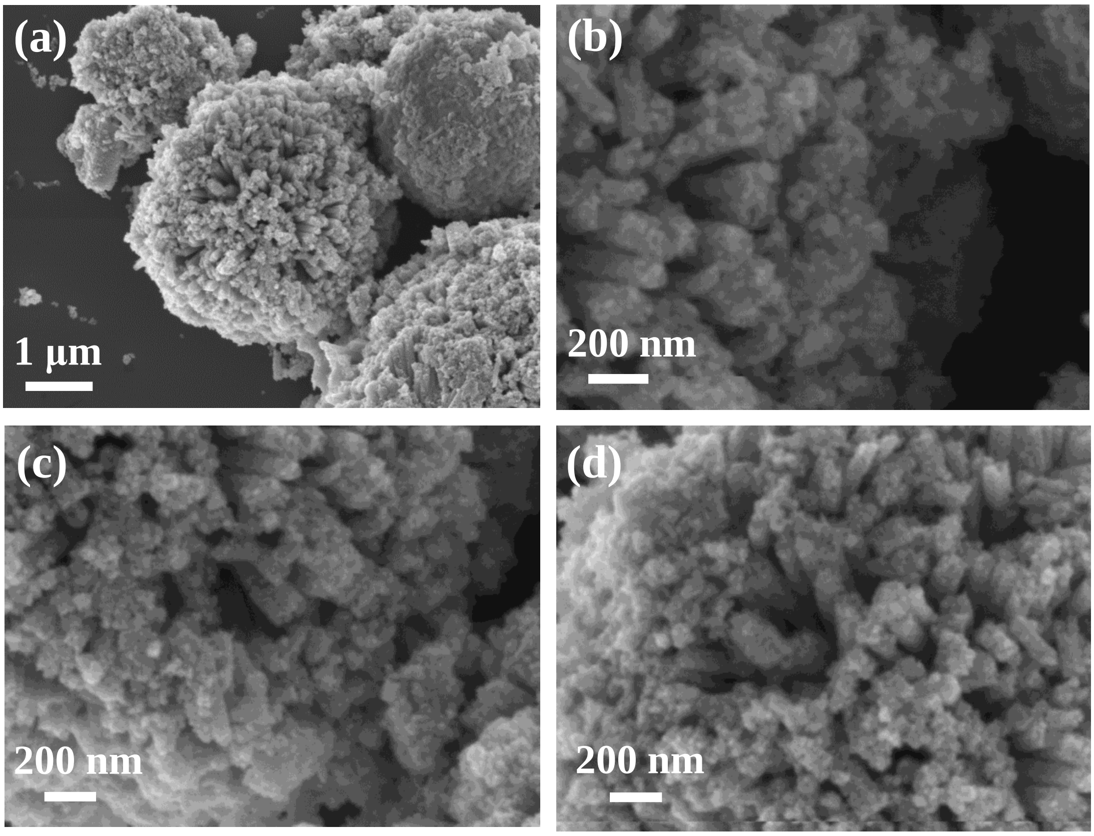

Figure S11. SEM images of $NiCo_2O_4$@CeO_2 electrodes: (a) at low magnification; (b-d) at different regions.

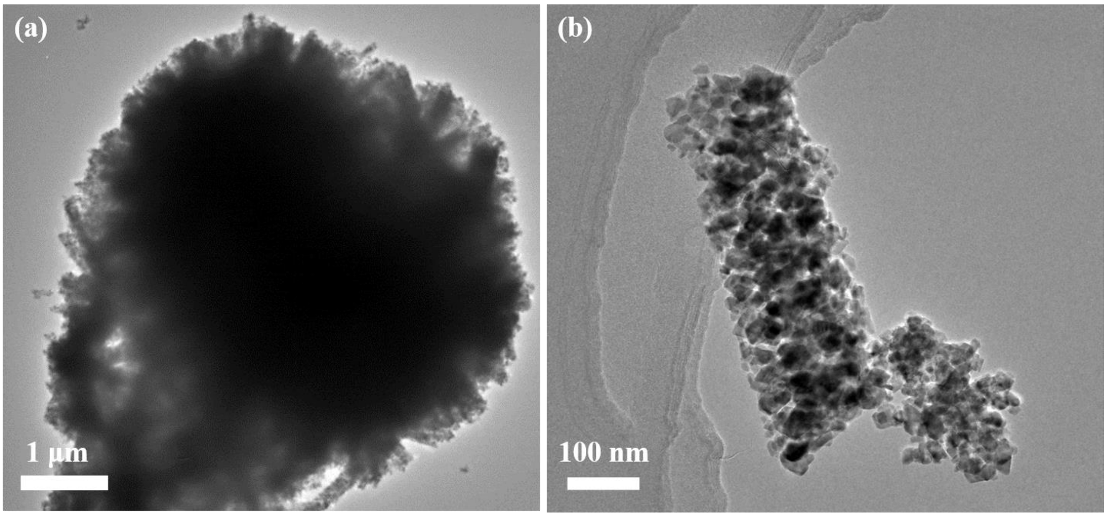

Figure S12. TEM of (a) the single urchin-like $NiCo_2O_4@CeO_2$ microsphere; (b) the single $NiCo_2O_4@CeO_2$ nanotube.

Table S1. The atomic content of $NiCo_2O_4@CeO_2$ derived from the EDX of SEM.

Element	Ni (wt%)	Co (wt%)	Ce (wt%)	O (wt%)
$NiCo_2O_4@CeO_2$	3.1	7.7	2.5	11.6

Table S2. Comparison of the catalyst in this work with other related $NiCo_2O_4$ or CeO_2 cathode materials for lithium oxygen batteries.

Catalytic materials	Discharge/Charge overpotential	Current density/Cycle number	Ref.
$NiCo_2O_4$@CeO_2	0.18/0.89 V	500 mA g^{-1}/400	This work
NiO@$NiCo_2O_4$@Ni	N/A	200 mA g^{-1}/176	[18]
Co_3O_4@$NiCo_2O_4$	1.3 V	100 mA g^{-1}/225	[19]
RuO_2@$NiCo_2O_4$	0.945 V	400 mA g^{-1}/128	[20]
$NiCo_2O_4$@BCNNT	0.97 V	100 mA g^{-1}/320	[21]
CeO_x/MC-X	0.24/1.41 V	100 mA g^{-1}/55	[22]
CeO_2/C	0.32/1.1 V	100 mA g^{-1}/440	[23]
CeO_2/δ-MnO_2	0.09/1.11 V	100 mA g^{-1}/296	[24]
MnO_2-CeO_2/CNT	0.2/0.68 V	100 mA g^{-1}/70	[25]

References

[1] W. Zhang, Y. Su, X. Zhang, Y. Yang, X. Guo, *RSC Advances* **2016**, 6, 64626.

[2] Z. Li, S. a. Liu, L. Li, W. Qi, W. Lai, L. Li, X. Zhao, Y. Zhang, W. Zhang, *Solar Energy Materials and Solar Cells* **2021**, 220, 110859.

[3] Q. Wang, L. Jiao, H. Du, Y. Si, Y. Wang, H. Yuan, *Journal of Materials Chemistry* **2012**, 22, 21387.

[4] J. Hao, S. Peng, H. Li, S. Dang, T. Qin, Y. Wen, J. Huang, F. Ma, D. Gao, F. Li, G. Cao, *Journal of Materials Chemistry A* **2018**, 6, 16094.

[5] S. Yang, Y. Liu, Y. Hao, X. Yang, W. A. Goddard, 3rd, X. L. Zhang, B. Cao, *Adv Sci (Weinh)* **2018**, 5, 1700659.

[6] T. Yi, L. Shi, X. Han, F. Wang, Y. Zhu, Y. Xie, *Energy & Environmental Materials* **2020**, 4, 586.

[7] Q. Zhang, Y. Liu, H. Lu, D. Tang, C. Ouyang, L. Zhang, *Electrochimica Acta* **2016**, 189, 147.

[8] H. Xing, G. Long, J. Zheng, H. Zhao, Y. Zong, X. Li, Y. Wang, X. Zhu, M. Zhang, X. Zheng, *Electrochimica Acta* **2020**, 337, 135817.

[9] Z. Cui, D. Zhang, J. Hu, C. Fang, *Journal of Alloys and Compounds* **2021**, 885, 160961.

[10] Y. Ouyang, R. Huang, X. Xia, H. Ye, X. Jiao, L. Wang, W. Lei, Q. Hao, *Chemical Engineering Journal* **2019**, 355, 416.

[11] A. K. Das, U. N. Pan, V. Sharma, N. H. Kim, J. H. Lee, *Chemical Engineering Journal* **2021**, 417, 128019.

[12] G. Liu, W. Li, R. Bi, C. Atangana Etogo, X.-Y. Yu, L. Zhang, *ACS Catalysis* **2018**, 8, 1720.

[13] Y. Wang, Y. Zhang, Z. Liu, C. Xie, S. Feng, D. Liu, M. Shao, S. Wang, *Angew Chem Int Ed Engl* **2017**, 56, 5867.

[14] K. Adpakpang, S. M. Oh, D. A. Agyeman, X. Jin, N. Jarulertwathana, I. Y. Kim, T. Sarakonsri, Y.-M. Kang, S.-J. Hwang, *Advanced Functional Materials* **2018**, 28, 1707106.

[15] J. J. Xu, Z. W. Chang, Y. Wang, D. P. Liu, Y. Zhang, X. B. Zhang, *Adv Mater* **2016**, 28, 9620.

[16] Y.-S. Liu, X. Liu, S.-M. Xu, Y.-L. Bai, C. Ma, W.-L. Bai, X.-Y. Wu, X. Wei, K.-X. Wang, J.-S. Chen, *Journal of Materials Chemistry A* **2019**, 7, 24524.

[17] W. H. Choi, B. C. Moon, D. G. Park, J. W. Choi, K. H. Kim, J. S. Shin, M. G. Kim, K. M. Choi, J. K. Kang, *Adv Sci (Weinh)* **2020**, 7, 2000283.

[18] H. Wang, B. Fan, Z. Luo, Q. Wu, X. Zhou, F. Wang, *Catalysis Science & Technology* **2021**, 11, 7632.

[19] J. Li, Y. Deng, L. Leng, M. Liu, L. Huang, X. Tian, H. Song, X. Lu, S. Liao, *Journal of Power Sources* **2020**, 450, 227725.

[20] L. Zou, Y. Jiang, J. Cheng, Y. Chen, B. Chi, J. Pu, L. Jian, *Electrochimica Acta* **2018**, 262, 97.

[21] X. Li, J. Liu, J. Zhang, S. Wu, D. Zhang, J. Shui, *Chemical Engineering Journal* **2021**, 411, 128403.

[22] L. Wang, S. Chen, J. Hei, R. Gao, L. Liu, L. Su, G. Li, Z. Chen, *Nano Energy*

2020, 71, 104570.

[23] Y. Hou, J. Wang, J. Liu, C. Hou, Z. Xiu, Y. Fan, L. Zhao, Y. Zhai, H. Li, J. Zeng, X. Gao, S. Zhou, D. Li, Y. Li, F. Dang, K. Liang, P. Chen, C. Li, D. Zhao, B. Kong, *Advanced Energy Materials* **2019**, 9, 1901751.

[24] C. Cao, J. Xie, S. Zhang, B. Pan, G. Cao, X. Zhao, *Journal of Materials Chemistry A* **2017**, 5, 6747.

[25] M. Salehi, Z. Shariatinia, *Electrochimica Acta* **2016**, 222, 821.
